# Cytokine response in asymptomatic and symptomatic *Plasmodium falciparum* infections in children in a rural area of south-eastern Gabon

**DOI:** 10.1371/journal.pone.0280818

**Published:** 2023-02-14

**Authors:** Sandrine Lydie Oyegue-Liabagui, Chérone Nancy Mbani Mpega Ntigui, Mérédith Flore Ada Mengome, Lady Charlene Kouna, Nathalie Pernelle Tsafack Tegomo, Neil Michel Longo Pendy, Jean-Bernard Lekana-Douki

**Affiliations:** 1 Unité d’Evolution Epidémiologie et Résistances Parasitaires (UNEEREP), Centre Interdisciplinaire de Recherches Médicales de Franceville (CIRMF), Franceville, Gabon; 2 Ecole Doctorale Régionale d’Afrique Centrale en Infectiologie Tropicale (ECODRAC), Université des Sciences et Techniques de Masuku, Franceville, Gabon; 3 Département de Biologie, Faculté des Sciences, Université des Sciences et Techniques de Masuku (USTM), Franceville, Gabon; 4 Unité Ecologie des Systèmes Vectoriels, Centre Interdisciplinaire de Recherches Médicales de Franceville (CIRMF), Franceville, Gabon; 5 Département de Parasitologie-Mycologie, Université des Sciences de la Santé (USS), Libreville, Gabon; Institut de recherche pour le developpement, FRANCE

## Abstract

*Plasmodium falciparum* is a parasite that causes asymptomatic or symptomatic malaria infections in humans depending on various factors. These infections are also a major cause of anemia in intertropical countries such as Gabon. Past studies have clearly demonstrated that inflammatory markers such as cytokines play a key role in the pathogenesis of malaria disease. However, the clinical manifestations of severe malaria vary according to the level of transmission and more information is needed to gain a better understanding of the factors involved. As such, the objective of this study was to investigate the circulating levels of nine cytokines in asymptomatic and symptomatic *P*. *falciparum* infections in Gabonese children and their roles in the pathogenesis of anemia. Blood samples were collected from 241 children aged 3 to 180 months in Lastourville, south-eastern Gabon. Diagnosis of *P*. *falciparum* infection was performed using Rapid Diagnosis Tests, microscopy and nested PCR. Levels in the plasma of the Th1 (IFN-γ, TNF-α, IL-6 and IL-12p70), Th17 (IL-17A and IL-22) and Th2 (IL-10, IL-4 and IL-13) cytokines were measured by ELISA. Data showed that IL-6, IFN-γ, IL-12p70, IL-10, and IL-13 levels were significantly higher in children with symptomatic *P*. *falciparum* infection compared to uninfected children. IL-10 levels were significantly higher in symptomatic children than in asymptomatic children, who had moderately increased levels compared to uninfected controls. Moreover, only IL-10 and IL-6 levels were significantly higher in children with severe malarial anemia compared to children with uncomplicated malaria who had significantly lower IL-10 levels than children with moderate malarial anemia. These data indicate that the progression of *P*. *falciparum* infection towards an advanced stage in children is accompanied by a significant increase in type Th1 and/or Th2 cytokines. These inflammatory mediators could serve as potential predictors of anemia for malaria patients.

## Introduction

*Plasmodium falciparum* is a parasite transmitted to people through the bite of an infected female *Anopheles* mosquito. The resulting infection can cause asymptomatic or symptomatic malaria infections. The World Health Organization (WHO) estimated that there were 14 million more malaria cases and 47,000 more deaths in 2020 than in 2019 [1]. In 2020, 229 million clinical malaria episodes and 409,000 deaths were recorded worldwide, the majority of which were among children in sub-Saharan Africa [2]. The clinical outcome of African children with malaria ranges from asymptomatic cases to severe forms of the disease or death. The outcome depends on many factors such as the type of parasite, host variability, socio-demographic aspects [3], and the immune response of hosts is a key determinant.

Although the immunopathological basis for the development of severe forms of malaria infection is not well understood, previous studies have demonstrated that cytokines play an important role [3–5]. Cytokines, mediators of the immune system, have been shown to be correlated with the development of symptomatic disease during *P*. *falciparum* infection [6–8]. They are involved in the protection of the body depending on their regulatory effects on different innate and adaptive cells in the host defense or in the immunopathology through excessive stimulation of their naturally useful pathways [9–12]. An early increase in Th1 cytokines has been associated with protection against *Plasmodium* however it has been observed a shift from Th1 to Th2 during pick of parasite levels for parasite clearance [4, 13, 14]. Both early and later uncontrolled cytokine levels have been associated with the development of immunopathology often found in severe forms of the disease [15, 16]. In addition, in order to restore homeostatic balance, cytokines with immunoregulatory activities, notably IL-10 and TGF-β, regulate the levels of Th1 and Th2 inflammatory mediators [17], which could be problematic in itself. Indeed, in the case of non-resumption of infection by an effective pro-inflammatory response, this would lead to the notions of immune tolerance and reservoir which would maintain the transmission of the parasite. In a previous work conducted in Gabon, we showed that IL-10 may be a better indicator of asymptomatic infection, as significantly higher levels of IL-10 were associated with asymptomatic forms of malaria in urban, semi-urban and rural areas [18]. We also observed previously significantly higher levels of IFN-γ, IL-6, IL-10, IL-13 in infected *P*. *falciparum* febrile children than in uninfected controls and found that, during severe malarial anemia, IL-6 and IL-10 could play a role in inflammatory response and pathophysiology [19].

The aim of the present study was to investigate and compare the Th1, Th2 and Th17 inflammatory cytokine responses in asymptomatic and symptomatic children infected with *P*. *falciparum* in south-eastern Gabon. We explore the hypothesis that an inflammatory marker can make possible the discrimination between an asymptomatic and a symptomatic infection and between different clinical manifestations of malaria among symptomatic individuals.

## Materials and methods

### Study site and ethical statement

The study took place in Lastourville, the capital of the Mulundu department, a rural area of south-eastern Gabon. The Gabonese National Research Ethics Committee approved this study (N°0023/2013/SG/CNE). A written informed consent was obtained from the parents or guardians of all the children included in the study. Asymptomatic children were recruited in community settings during a voluntary screening campaign organized by the Centre Interdisciplinaire de Recherches Médicale de Franceville (CIRMF) during three days. The symptomatic children were enrolled in the medical center of Lastourville, where it has been shown that the prevalence of plasmodial infection is 79% [20].

### Study design

#### Participants

A total of 425 children aged 3–180 months were enrolled in the present study. Children were classified in three groups: uninfected children (healthy controls), asymptomatic or symptomatic children infected with *P*. *falciparum* after diagnosis. An infected participant was considered to be any child who had a positive diagnosis for Plasmodium by either Rapid Diagnostic Testing or microscopy and whose PCR identified *Plasmodium falciparum* as the species causing the infection.

An asymptomatic *P*. *falciparum* infection was defined by the absence of a febrile syndrome or a history of fever (temperature ≥ 37.5°C) during the two weeks before sampling. Children coming to the medical center with a febrile syndrome or a history of fever in the past 48 h were classified in the symptomatic group. Only children infected with *P*. *falciparum* were included in the study. Children with known chronic diseases like sickle cell disease and HIV or others infections were not included in this study. The group of symptomatic children infected with *P*. *falciparum* was then divided into three groups according to the hemoglobin concentration in line with the 2014 criteria [21]: uncomplicated malaria (hemoglobin >10 g/dl), moderate malarial anemia (hemoglobin between 5–10 g/dl) and severe malarial anemia (hemoglobin ≤ 5 g/dl) in children. A treatment of Artemether/Lumefantrine was given to all the children infected with *Plasmodium*.

#### Hematologic analysis

COBAS ABX (Roche) counters were used for the hematologic analysis.

The rest of the whole blood was immediately centrifuged to separate the plasma from the red blood cells. The plasma was then placed at—80°C and the pellet was used for DNA extraction.

#### Malaria diagnosis

Three different tests were used: (i) Rapid Diagnostic Testing (RDT) (OptiMAL-IT® test (HRP2)) to determine the presence of *Plasmodium* parasites, (ii) microscopy to determine the parasitemia load, (iii) and nested PCR to determine the *Plasmodium* species. Briefly, according to the Lambarene method [22], thick blood smears from 10 μL samples were stained with 10% Giemsa. After washing and drying, the number of *Plasmodium* parasites was determined only for the asymptomatic participants by two qualified technicians from the CIRMF, Gabon.

#### Identification of *Plasmodium* species

DNA (5 μl) was extracted from all children diagnosed positive for *Plasmodium* by microscopy and/or RDT with the Qiagen kit (QIAamp DNA Mini kit; Qiagen, Hilden, Germany) and then amplified with 1× Taq polymerase buffer (Invitrogen, San Diego, California, USA). 0.8 μM for each of the primers rPLU5 and rPLU6 for the first PCR reaction, and rFAL1, rFAL2, rMAL1, rMAL2, rVIV1, rVIV2, rOVA1 and rOVA2 for the nested PCR (Eurogentec, Seraing, Belgium) [23] with 0.2 mM dNTP, 2 mM MgCl2 and 0.024 U of Taq DNA polymerase (Invitrogen) were used [20]. The products of the nested PCR were then analyzed by electrophoresis on 2% agarose gel.

#### Cytokine measurements

An enzyme-linked immunosorbent assay (ELISA) was used to detect human cytokines IL-6, TNF-α, IFN-γ, IL-17A, IL-22, IL-12 p70, IL-4, IL-13 and IL-10 in plasma according to the manufacturer’s instructions (Bender MedSystems, Vienna, Austria). Optical densities (OD) were measured at 450 nm with a reference at 620 nm using an ELISA plate reader (Stat Fax 3200®; Bioblock/Fisher Scientific, Vienna, Austria). Each sample was tested in duplicate, and the mean of the OD values was used for analysis. The detection limits were: 2 pg/ml for IL-10, IL-4 and IL-6; 4 pg/ml for TNF-α, IFN-γ, IL-12p70, IL-17A and IL-13 and 8 pg/ml for IL-22. During statistical analysis all values below limit of detection were replaced by the value of limit of detection for each cytokine.

### Statistical analysis

SPSS version 17.0 for Windows (SPSS Inc., Chicago, USA) and GraphPad Prism software version 8.4 were used for statistical analysis. The normality of the data was determined using the Kolmogorov-Smirnorv tests. Then, as the data did not follow a normal distribution, statistically significant differences between the three groups were analyzed with the non-parametric Kruskal-Wallis test for each cytokine and when this comparison revealed a significant difference, the Mann-Whitney *U* test was used for pairwise comparisons. A multivariate analysis was also performed using the Multivariate Spearman correlation test. All *p* values < 0.05 were statistically significant.

## Results

### Socio-demographic and clinical characteristics of the participants

Of the 425 enrolled-children, 241 were included in this study and among them, 44 were infected with *P*. *falciparum* but asymptomatic, 127 had a *P*. *falciparum* infection and were symptomatic, and 70 were uninfected (control group). The demographic and clinical characteristics of the participants according to their status (symptomatic, asymptomatic and uninfected) are presented in **Table 1**. The sex ratio (male/female) was slightly in favor of males in symptomatic children (1.43), of females in the asymptomatic group (0.76), but the ratio was 1 in uninfected children. The median age in the symptomatic group of children was 24 (12–38) months, and it was significantly lower than in the asymptomatic and uninfected groups, 72 (44–139) versus 84 (48–120) months, respectively (*p* <0.001). Naturally, the body temperature of symptomatic children was significantly higher than that of asymptomatic and uninfected children [38.0°C (37.0–38.9°C) vs. 36.4°C (35.7–36.8°C) vs. 36.4°C (36.1–36.7°C), respectively (*p* <0.001)]. In the group of symptomatic children, the platelet and red blood cell counts [134 (91–203) 10^3^ cells/mm^3^ and 3.1 (2.5–3.7) 10^6^ cells/mm^3^, respectively] and the hemoglobin concentration [7.1 (6.0–8.7) g/dL] were below normal values [(150–400)10^3^ cells/mm^3^ for platelets, (3.8–6.0) 10^6^ cells/mm^3^ for red blood cells and (11.5–17.0) g/dL for hemoglobin]. However, there was no disturbance of the white blood cell level [8.5 (6.4–11.9) 10^3^ cells/mm^3^; normal range values (3.5–10.0) 10^3^ cells/mm^3^].

**Table 1:** Demographical and clinical parameters of the children included in the study.

Parameters	Children			
	Uninfected	Asymptomatic	Symptomatic	*P*
	N = 70	N = 44	N = 127	
Sex ratio	0.79	0.69	2.31	
Age (months) (IQR)	80 (46–124)	74 (47–146)	24 (12–36)	<0.001
Temperature (IQR)	36.4 (36.0–36.7)	36.4 (35.7–36.8)	38.0 (37.0–38.9)	<0.001
White blood cells (10^3^ cells/mm^3^) (IQR)	ND	ND	8.5 (6.4–11.9)	-
Red blood cells (10^6^ cells/mm^3^) (IQR)	ND	ND	3.1 (2.5–3.7)	-
Hemoglobin (g/dL) (IQR)	ND	ND	7.1 (6.0–8.7)	-
Platelets (10^3^ cells/mm^3^) (IQR))	ND	ND	134 (91–203)	-
Parasitemia (parasites/μL) (IQR)	ND	2520 (1120–5600)	ND	-

Sex ratio, age, temperature, hemoglobin level, platelets, white and red blood cell counts and parasite load in respectively all children (symptomatic and asymptomatic children infected with *P*. *falciparum*, and uninfected children). The uninfected children were exposed to *P*. *falciparum* but negative for parasites in thick blood smears and/or in rapid detection test kits. IQR, interquartile range; ND, not determined. Significant levels were calculated using the Kruskal-Wallis test and was defined from *p* < 0.05.

### Higher IL-6, IFN-γ and IL-12p70 levels in children with symptomatic *P*. *falciparum* infection

Th1, Th17 and Th2 cytokine levels in the plasma samples were assessed. The levels of nine cytokines measured in plasma revealed an increase of IL-6, IFNg, IL-22 and IL12p70 in children with symptomatic malaria compared to uninfected children.

**Fig 1:**
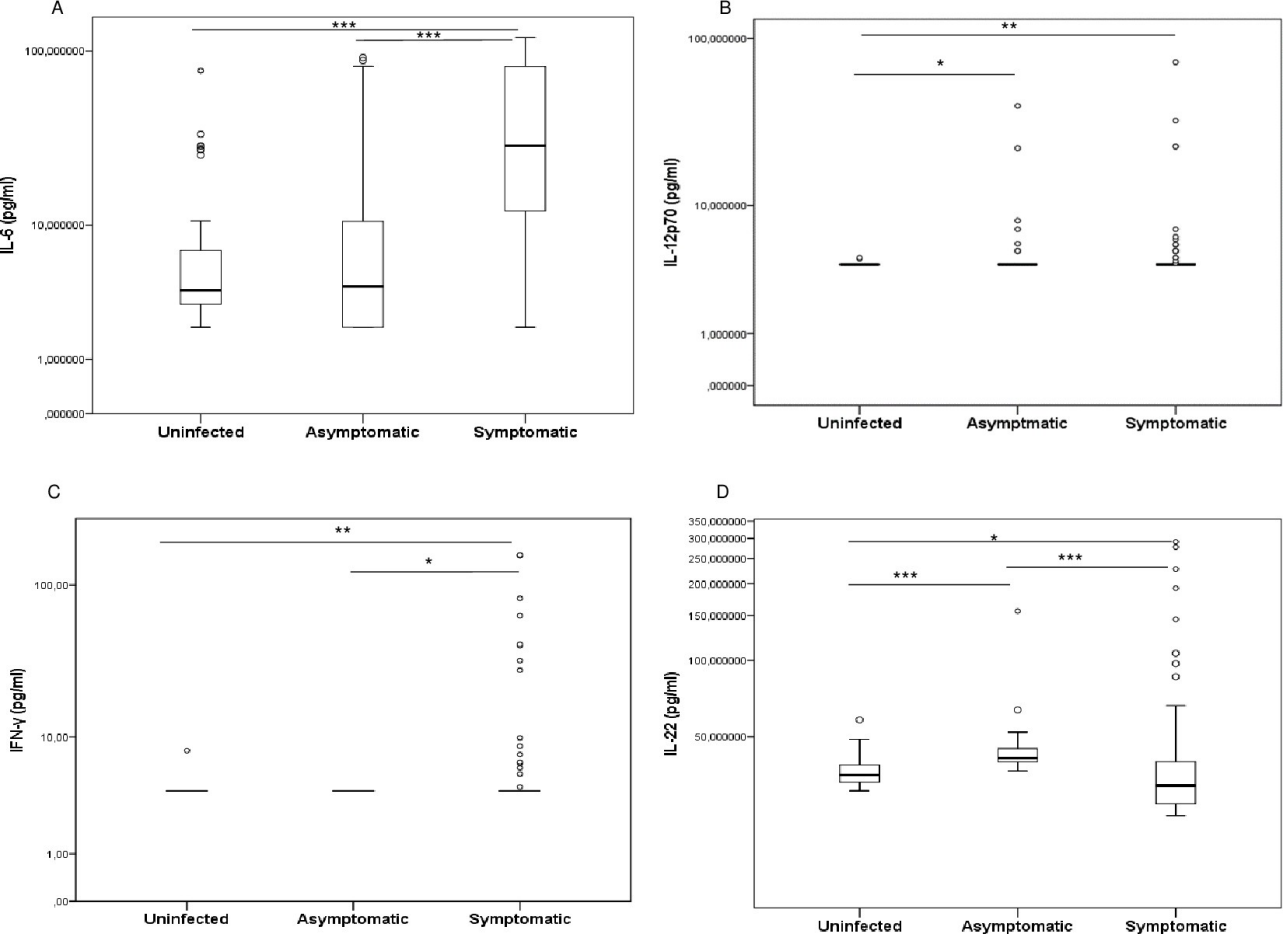
Levels of interleukin IL-6 (A), IL-12p70 (B), IFN-γ (C) and IL-22 (D) in plasma in children infected with *P*. *falciparum* and uninfected children. Concentrations were quantified using enzyme-linked immunosorbent assays (ELISA) and compared between uninfected, symptomatic and asymptomatic children in pairs. Cytokine concentrations were represented by boxplots with medians and interquartile range (IQR) in pg/ml on the log10 scale. Statistically significant differences between groups were tested using the Mann–Whitney method and are represented as *p < 0.05; **p < 0.001; ***p = 0.0001; ****p < 0.0001.

Indeed, IL-6 and IFN-γ were found significantly higher levels in symptomatic children compared to asymptomatic-*P*. *falciparum* infected and uninfected children [29.36 (12.09–81.82) pg/ml vs. 4.04 (2.00–10.55) pg/ml; *p* < 0.001 and 29.36 (12.09–81.82) pg/ml vs. 3.81 (2.96–7.00) pg/ml; *p* < 0.001 for IL-6 and 4.00 (4.00–4.00) pg/ml vs. 4.00 (4.00–4.00) pg/ml; *p* = 0.013 and 4.00 (4.00–4.00) pg/ml vs. 4.00 (4.00–4.00) pg/ml; *p* = 0.002 for IFN-γ, respectively]. However, asymptomatic and uninfected children had comparable levels for these two cytokines (*p* = 0.783 and *p* = 0.472, respectively) (**Fig 1A and 1C**).

Furthermore, IL-22 was significantly higher in asymptomatic children compared to uninfected children [41.17 (39.59–45.14) pg/ml vs. 35.21 (32.90–38.78) pg/ml; *p* < 0.001]. Strangely, symptomatic children had significantly lower levels of IL-22 than asymptomatic- *P*. *falciparum* infected and uninfected children [31.95 (26.95–39.89) pg/ml vs. 41.17 (39.59–45.14) pg/ml; *p* < 0.001 and 31.95 (26.95–39.89) pg/ml vs. 35.21 (32.90–38.78) pg/ml; *p* = 0.015] (**Fig 1D)**.

However, IL-12p70 levels were significantly higher in symptomatic and asymptomatic children compared to uninfected children [4.00 (4.00–4.00) pg/ml vs. 4.00 (4.00–4.00) pg/ml; *p* = 0.006 and 4.00 (4.00–4.00) pg/ml vs. 4.00 (4.00–4.00) pg/ml; *p* = 0.013]. Moreover, IL-12p70 levels in asymptomatic children were comparable to that of symptomatic children (*p* = 0.942) (**Fig 1B)**. In other hand, the pairwise comparisons between these groups revealed no differences for TNF-α and IL-17A (*p* > 0.05; S1 Fig).

### Higher levels of IL-10 and IL-13 during symptomatic infection

**Fig 2:**
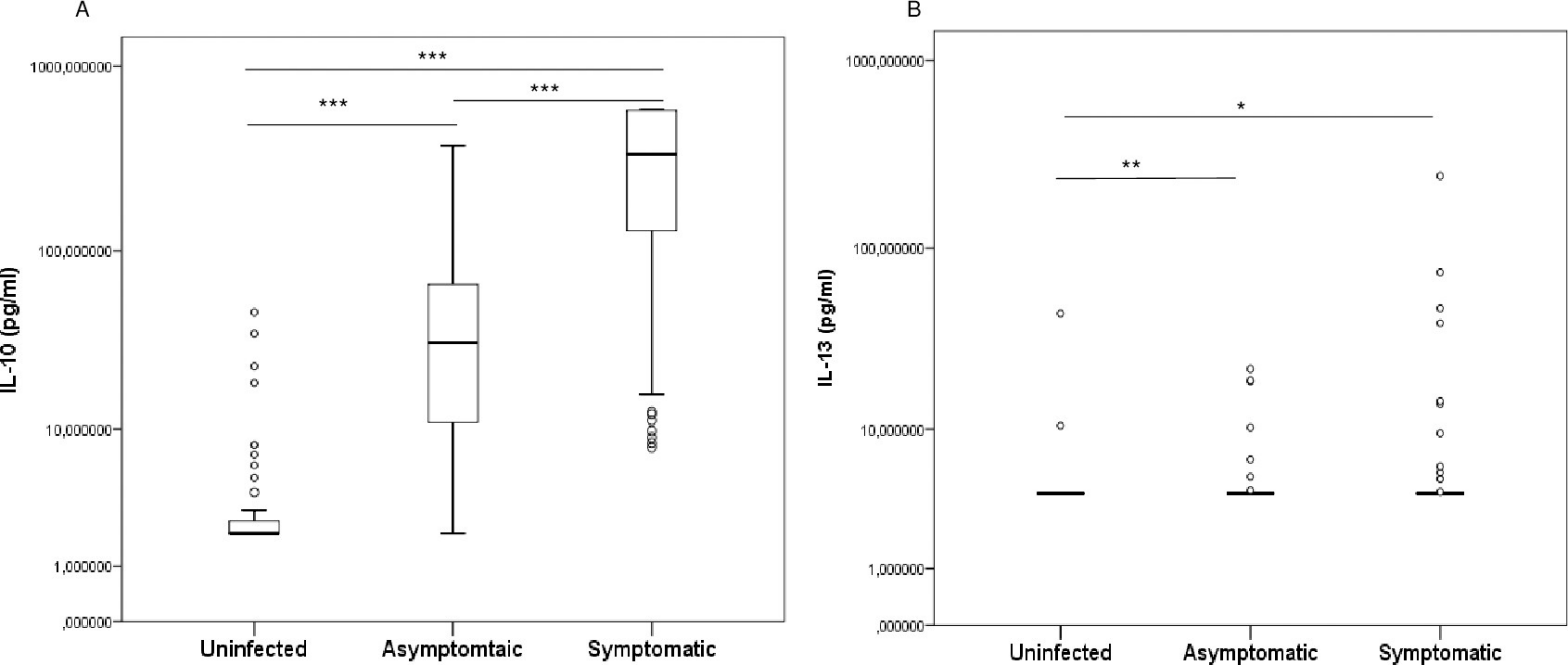
Levels of IL-10 (A) and IL-13 (B) in plasma in children infected with *P*. *falciparum* and uninfected children. Concentrations were quantified using enzyme-linked immunosorbent assays (ELISA) and compared between uninfected, symptomatic and asymptomatic children in pairs. Cytokine concentrations were represented by boxplots with median and interquartile range (IQR) in pg/ml on the log10 scale. Statistically significant differences between groups were tested using the Mann–Whitney method and are represented as *p < 0.05; **p < 0.001; ***p = 0.0001; ****p < 0.0001.

IL-10 levels were significantly higher in symptomatic children compared to asymptomatic children [335.13 (128.20–579.00) pg/ml vs. 31.15 (10.95–65.54) pg/ml; *p* < 0.0001] who had significantly higher levels than uninfected children [31.15 (10.95–65.54) pg/ml vs. 0 (0–2.50) pg/ml; *p* < 0.0001] (**Fig 2A**).

For IL-13, higher levels were observed in symptomatic and asymptomatic children compared to the uninfected [4.00 (4.00–4.00) pg/ml vs. 4.00 (4.00–4.00) pg/ml; *p* = 0.030 and 4.00 (4.00–4.00) pg/ml vs. 4.00 (4.00–4.00) pg/ml; *p* = 0.006, respectively] (**Fig 2B**). However IL-13 levels were comparable between symptomatic and asymptomatic children (*p* = 0.371). Also, comparisons between groups by pair showed no differences for IL-4 (*p* > 0.05; S1 Fig).

### Higher IL-10 and IL-6 levels in severe malarial anemia cases

**Fig 3:**
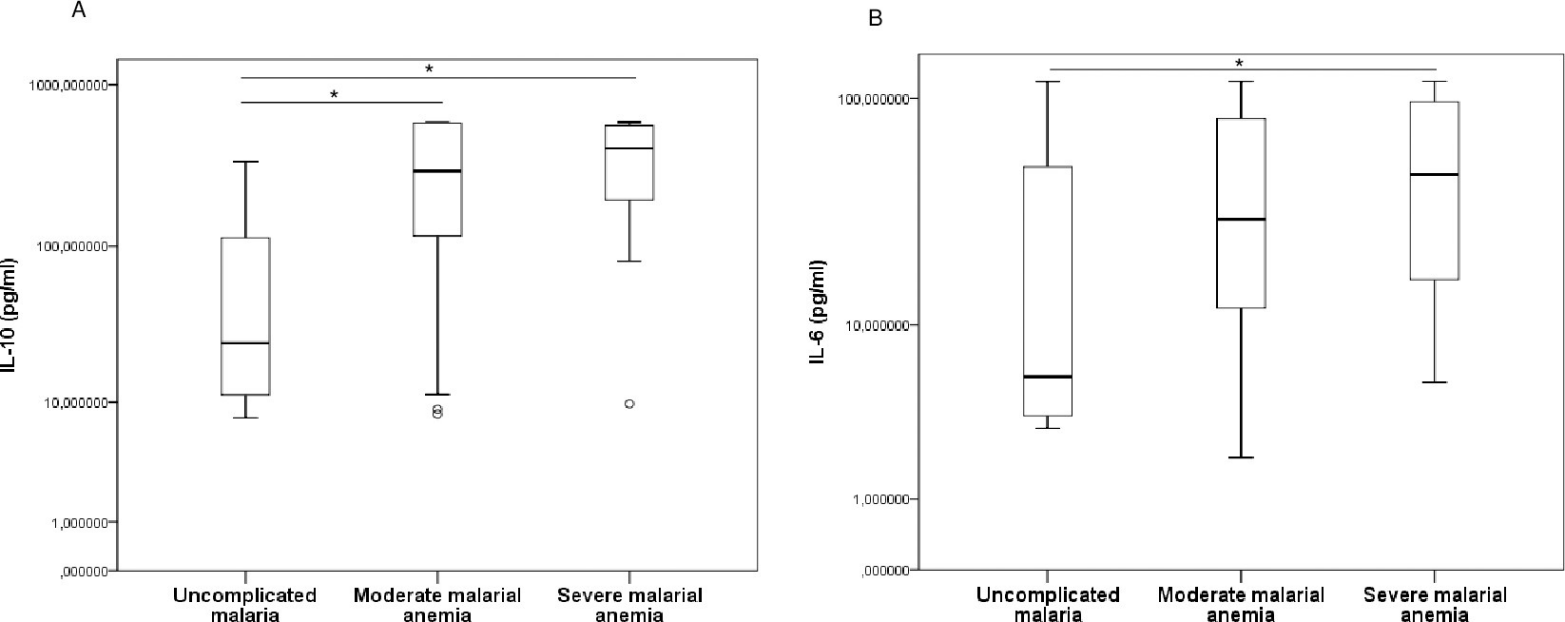
Levels of IL-10 (A) and IL-6 (B) in plasma in children with symptomatic uncomplicated malaria, moderate malarial anemia and severe malarial anemia. Concentrations were quantified using enzyme-linked immunosorbent assays (ELISA) and compared between the different groups in pairs. Cytokine concentrations were represented by boxplots with medians and interquartile range (IQR) in pg/ml on the log10 scale. Statistically significant differences between groups were tested using the Mann–Whitney method and are represented as *p < 0.05; **p < 0.001; ***p = 0.0001; ****p < 0.0001.

The symptomatic group was subdivided into three groups according to the hemoglobin concentration of the 184 participants who had hematological parameters: uncomplicated malaria (n = 11), moderate malarial anemia (n = 144) and severe malarial anemia (n = 29) (**Fig 3**). The comparison of these three groups show significant differences for IL-10, IL-6, IFN-γ and IL-22 (*p* < 0.001, *p* < 0.001, *p* = 0.001 and *p* = 0.002, respectively) and no differences for TNF-α, IL-4, IL-13, IL-17A and IL-12p70 (*p* > 0.05) (Data not showed).

Levels of IL-10 were significantly higher in severe and moderate malarial anemia compared to the uncomplicated malaria group [395.80 (186.10–563.40) pg/ml vs. 26.58 (9.79–260.70) pg/ml; *p* = 0.014 and 293.38 (115.30–579.69) pg/ml vs. 26.58 (9.79–260.70) pg/ml; *p* = 0.031, respectively]. The moderate and severe malarial anemia groups revealed no statistical difference in their levels (*p* = 0.317) (**Fig 3A**).

In addition, IL-6 levels in the severe malarial anemia group were significantly higher than in the uncomplicated malaria group [36.03 (15.36–97.86) pg/ml vs. 5.99 (3.50–93.98) pg/ml; *p* = 0.042] (**Fig 3B**). Comparison between the uncomplicated malaria group and moderate malarial anemia group, and between moderate and severe malarial anemia groups revealed no statistical difference for IL-6 (*p* = 0.145 and *p* = 0.167, respectively). Otherwise the pairwise comparisons between groups revealed no differences for IFN-γ, TNF-α, IL-17A, IL-22, IL-12p70, IL-4 and IL-13 (*p* > 0.05; S2 Fig).

### Association between cytokine level, age and parasitemia in asymptomatic *P*. *falciparum* infected children

The effect of age and parasite load on cytokine levels was assessed using a Spearman multivariate analysis. None of the cytokine levels were associated with age however, only IL-6 was positively correlate with parasitemia (r = 0.38, *p* = 0.030). The levels of several cytokines were correlated with each other (**Table 2**). IL-12p70 was associated with IFN-γ, TNF-α, IL-4, IL-13, IL-17A and IL-22 (r = 0.48, *p* = 0.008; r = 0. 82, *p* < 0.0001; r = 0.97, *p* = 0.0001; r = 0.44, *p* = 0.013; r = 0.86, *p* < 0.0001 and r = 0.86, *p* < 0.0001, respectively). There was a positive association between TNF-α and each of the following five cytokines: IL-10, IL-4, IL-13, IL-17A and IL-22 (r = 0.43, *p* = 0.043; r = 0.99, *p* = 0.0001; r = 0.41, *p* = 0.024; r = 0.99, *p* = 0.0001 and r = 0.54, *p* = 0.002, respectively). IL-4 in turn was positively associated with IL-13, IL-17A and IL-22 (r = 0.52, *p* = 0.004; r = 0.99, *p* = 0.0001 and r = 0.97, *p* = 0.0001, respectively) and IL-13 positively associated with IL-22 (r = 0.56, *p* = 0.001). In addition, IL-17A was significantly associated with IL-22 (r = 0.59, *p* < 0.001, respectively).

**Table 2:** Multivariate bilateral analysis between cytokines, age and parasitemia in asymptomatic children.

	Age	Parasitemia	IFN-γ	TNF-α	IL-10	IL-6	IL-4	IL-13	IL-12p70	IL-17A	IL-22
Age	1										
Parasitemia	0.11	1									
IFN-γ	-0.32	-0.01	1								
TNF-α	0.32	-0.08	-0.05	1							
IL-10	-0.15	0.06	-0.04	**0.43** *	1						
IL-6	-0.30	**0.38** *	-0.16	-0.07	0.21	1					
IL-4	0.21	-0.07		**0.99** ***		-0.05	1				
IL-13	-0.03	-0.17		**0.41** *	-0.17	-0.17	**0.52** **	1			
IL-12p70	0.26	-0.01	**0.48** **	**0.82** ****	0.15	-0.01	**0.97** ***	**0.44** *	1		
IL-17A	0.31	-0.08		**0.99** ***		-0.05	**0.99** ***	0.34	**0.86** ****	1	
IL-22	0.14	-0.09		**0.54** **	-0.16	-0.04	**0.97** ***	**0.56** **	**0.86** ****	**0.59** ***	1

A multivariate Spearman correlation analysis was performed for cytokines, parasitemia and age in asymptomatic children infected with *P*. *falciparum*. Data on temperature and age which were found to be irrelevant have not been presented in this table. Correlation coefficient is given and is strong for rho = 0.7 to 1, moderate for rho = 0.5 to 0.7 and weak for rho = 0.2 to 0.5 and for a value close to 0 there is not a correlation. The statistically significant differences are presented in bold and denoted by

*p < 0.05

**p < 0.01

***p < 0.001

****p < 0.0001. Abbreviations: IL, interleukin; TNF, tumor necrosis factor.

### Association between cytokine levels, age and temperature in symptomatic *P*. *falciparum* infected children

Data revealed that IL-10 and IL-6 were associated with temperature (r = 0.57, *p* = 0.001 and r = 0.48, *p* = 0.009, respectively) and age was positively associated with IL-13 (r = 0.37, *p* = 0.001) (**Table 3**). Both IL-10 and IL-6 showed a moderate association (r = 0.69, *p* < 0.0001) and each of them was respectively weakly correlated with IFN-γ and TNF-α (r = 0.25, *p* = 0.017; and r = 0.23, *p* < 0.044, respectively). Otherwise IL-12p70 was positively correlated with IFN-γ, IL-13, IL-17A and IL-22 (r = 0.61, *p* < 0.0001; r = 0.86, *p* < 0.001; r = 0.89, *p* < 0.001 and r = 0.30, *p* = 0.027, respectively). TNF-α levels were associated with IL-22 and IL-13 (r = 0.53, *p* < 0.0001 and r = 0.44, *p* = 0.001, respectively). IL-17A levels were associated with TNF-α, IL-4, IL-13, and IL-22 (r = 0.49, *p* < 0.001; r = 0.54, *p* < 0.001; r = 0.28, *p =* 0.021 and r = 0.38, *p* = 0.001 respectively).

**Table 3:** Multivariate bilateral analysis between cytokines, age and temperature in symptomatic children.

	Temperature	Age	IFN-γ	TNF-α	IL-10	IL-6	IL-4	IL-13	IL-12p70	IL-17A	IL-22
Temperature	1										
Age	0.13	1									
IFN-γ	0.28	-0.20	1								
TNF-α	0.05	0.03	-0.04	1							
IL-10	**0.57** **	-0.05	**0.25** *	0.21	1						
IL-6	**0.48** **	-0.02	0.19	**0.23** *	**0.69** ****	1					
IL-4	0.03	0.03	0.22	0.30	-0.10	-0.02	1				
IL-13	0.06	**0.37** **	0.07	**0.53** ****	0.21	0.12	0.16	1			
IL-12p70	-0.01	-0.16	**0.61** ****	0.07	0.09	0.13	-0.05	**0.86** ***	1		
IL-17A	-0.31	0.09	-0.05	**0.49** ***	0.16	0.16	**0.54** ***	**0.28** *	**0.89** ***	1	
IL-22	0.09	-0.01	-0.04	**0.44** **	0.19	0.17	0.18	0.10	**0.30** *	**0.38** **	1

The multivariate Spearman correlation analysis was carried out between cytokines, temperature and age in children with a symptomatic *P*. *falciparum* infection. Correlation coefficient is given and is strong for rho = 0.7 to 1, moderate for rho = 0.5 to 0.7 and weak for rho = 0.2 to 0.5 and for a value close to 0 there is not a correlation. The statistically significant differences are presented in bold and denoted by

*p < 0.05

**p < 0.01

***p < 0.001

****p < 0.0001. Abbreviations: IFN, interferon; IL, interleukin; TNF, tumor necrosis factor.

## Discussion

This study investigated the inflammatory cytokine levels in children with *P*. *falciparum* asymptomatic and symptomatic infection and in uninfected children living in the rural area of Lastourville in south-eastern Gabon.

Symptomatic children were significantly younger than asymptomatic and uninfected children. Other studies also found that children with asymptomatic malaria infection were older than children with symptomatic malaria infection [24, 25]. Indeed, in areas of high transmission, children frequently infected with *Plasmodium* species acquire immunity with age, which leads to a decrease in the number of clinical malaria infections, which in turn is associated with the development of asymptomatic infections [26, 27]. In other hand, during erythrocytic phase the release of merozoites after each cycle creates episodes of fever. These are reflected in an increase in body temperature corresponding to the onset of clinical signs or symptoms.

Moreover, infection by *Plasmodium* targets and destroys red blood cells and consequently affects hematological parameters like red blood cell count, hemoglobin level and platelet count. Thrombocytopenia has been found in patients suffering from acute *P*. *falciparum* malaria [28]. The parasites also induce the destruction of uninfected red blood cells by releasing antigen on their membrane [29, 30], as well as the sequestration of platelets [31].

In this study, we found that symptomatic children with *P*. *falciparum* infection had significantly higher IL-6 and IFN-γ levels than asymptomatic and uninfected children. In addition, symptomatic and asymptomatic children also had significantly higher IL-12p70 and IL-13 levels and asymptomatic children, significantly higher IL-22 levels than uninfected children. However, IL-10 levels were significantly higher in symptomatic compared to asymptomatic children, who in turn had significantly higher levels than uninfected children. All of these suggesting that *P*. *falciparum* induces the Th1, Th2 and Th17 cytokines. Moreover in the present study, the positive correlations between several cytokines in asymptomatic and symptomatic *P*. *falciparum* infection suggests that these cytokines are produced concurrently. Indeed, protection mediated by pro-inflammatory response may be linked to the Th1 and Th17 cytokines responses that are associated with early immune mechanisms which control *Plasmodium* infection [32–34]. The intensity of Tregs and Th17 activation has also been shown to play an important role in regulating the maturation and function of dendritic cells in the early stages of *Plasmodium* infection, which is a key factor in the outcome of malaria infection [35].

IL-6 is generated during acute malaria infection mainly by peripheral blood mononuclear cells [36] and have been reported significantly elevated in children with symptomatic malaria infections when compared to healthy controls [37]. This cytokine is up-regulated by TNF-α and acts in concert with other inflammatory mediators to limit parasitemia load [10, 38, 39]. IFN-γ is an important Th1 cytokine which can be released by both innate and adaptive immune cells including NK cells, γδT cells, CD4^+^ and CD8^+^T cells during *P*. *falciparum* infection [40] and can amplify the inflammatory response by increasing the production of TNF-α, IL-6, IL-12 and IL-1 by macrophages [41, 42]. It has been also reported that previous exposures to *Plasmodium* influenced the production of cytokines by adaptive immune cells (CD4+ T cells) [43] and attenuated the semi-innate Vδ2+ γδ T cells in response to malaria [40]. Also a decreased risk of clinical malaria once infected has been associated with the frequency of CD4 T cells producing IL-10 but not inflammatory cytokine like IFN-γ [44]. In addition, repeated malaria infection was associated with an altered proportion of Vδ2(+) γδ T cells and cytokine production in response to *Plasmodium* antigens, as well as increased expression of immunoregulatory genes [45]. This loss and dysfunction of Vd2+ T cells were statistically associated with the development of asymptomatic infection [45–47]. These support the fact that cytokine levels except for IL-10, IL-13, IL-12 and IL-22 are altered in asymptomatic children. During asymptomatic infection, IL-10 could act as a regulatory cytokine to maintain the balance Th1/ Th2 cytokines production and thus suppress inflammatory response and promote the preservation of parasites [5, 48]. The fact that symptomatic infected children have higher levels of IL-10 and still have symptoms, while the lower levels of this cytokine observed in the asymptomatic would suppress or decrease inflammation, could be also related to the anti-*Plasmodium* humoral immunity. Indeed, symptomatic children under the median age of 24 months have not yet been regularly exposed to *Plasmodium* or not exposed at all to develop natural immunity. Parasite invasion in the former would induce effector T-cell and macrophage/monocyte responses, leading to the release of greater amounts of cytokines [7, 49, 50]. It has been also showed that *Plasmodium falciparum* infection results in high levels of parasite-infected red blood cells that trigger systemic inflammation and fever in malaria-naïve individuals [51]. It was reported that during asymptomatic *P*. *falciparum* infection, the autoreactive polyclonal response associated with elevated IgG against MSP3 and high plasma levels of IL-10 and IFN-γ may contribute to host protective mechanisms during the immune response [52]. Also a continuous exposure to the *Plasmodium falciparum* causes an accumulation of specific memory responses. In addition, a recent study in experimental model have showed that the regulatory cytokine IL-10 plays an essential role in promoting germinal center B cell responses during malaria in early stage of infection [53]. Germinal centers being sites where high-affinity antibodies are generated from plasma cells and memory B cells during T cell–dependent immune responses [54, 55]. So the significantly higher IL-10 levels in symptomatic children compared to asymptomatic children, who in turn had significantly higher levels than uninfected children, show that IL-10 could be a good predictor of the progression of malaria infection.

Malaria infection causes paroxysmal fever which is triggered by strong pro-inflammatory responses involving pyrogenic cytokines such as IL-1β and TNF-α [56]. Surprisingly, in our study IL-6 and IL-10 were moderately associated with temperature and only Il-6 was correlated with parasitemia load. This result is similar to that of Hugosson et al. who found that in children with acute uncomplicated *P*. *falciparum* infection, body temperature was correlated with levels of IL-10, IFN-γ and IL-6, and parasitemia with the levels of IL-6 and IL-10 [57].

In addition, in order to determine the role played by cytokines in the development of anemia during *P*. *falciparum* infection, the symptomatic group was divided into three groups: uncomplicated malaria, moderate malarial anemia and severe malarial anemia. The significantly higher levels of IL-6 and IL-10 observed in severe malarial anemia than in uncomplicated malaria suggest that these cytokines are induced during *P*. *falciparum* malarial anemia and play a role in the pathophysiology [16, 19]. Indeed, after screening 186 papers, Perkins et al. stipulated that pathogenesis could largely be due to a dysregulation in cytokines, growth factors, chemokines and other effector molecules [58]. According to these authors, the mechanisms leading to the very low hemoglobin levels observed in children with severe malarial anemia are hemolysis and phagocytosis of parasitized and non-parasitized red blood cells. In addition, and to a large extent, the suppression of erythropoiesis induced by the deregulation of innate inflammatory mediators generated by PfHz is an important factor. In this case, severe malarial anemia would be a "chronic" state in which the ineffective pro-inflammatory immunological response against *P*. *falciparum* has been followed by the release of anti-inflammatory cytokines such as IL-10 to prevent inflammatory damage to the host. This suppression of erythropoiesis is reflected by anemia observed in our children.

The main limitations of this study are that the *Plasmodium* parasitemia load was not determined for all symptomatic participants enrolled in the hospital and some of the uninfected children may have been misclassified if they had a submicroscopic parasite load. In addition, asymptomatic infections might be, at least in part, pre-symptomatic infections, since no follow up was done to confirm the asymptomatic status. Also hematological analysis and intestinal parasite diagnosis were not performed in asymptomatic-uninfected and infected children. Despite these limitations, the present study was able to show that symptomatic *P*. *falciparum* infection is associated with increased levels of the cytokines IL-10, IL-13, IL-6, IFN-γ and IL-12p70 while asymptomatic infection is marked by increased levels of IL-10, IL-13, IL-22 and IL-12p70.

In conclusion, our results show that symptomatic *P*. *falciparum* infection in Gabonese children is associated with a more significant increase of some inflammatory Th1 and Th2 cytokine levels compared to asymptomatic children who, in turn, have significantly higher moderate levels than those observed in uninfected children. In view of our results, IL-10 might be a good marker of malaria pathogenesis as well as serves as an indicator of severe anemia during a malaria infection.

## Supporting information

S1 Fig Levels of TNF-α, IL-17A and IL-4 in plasma in children infected with *P*. *falciparum* and uninfected children. Circulating levels of each in the plasma were compared between uninfected, symptomatic and asymptomatic children in pairs. Concentrations were quantified using enzyme-linked immunosorbent assays (ELISA). Cytokine concentrations were represented by boxplots with median and interquartile range (IQR) in pg/mL on the log10 scale. Statistically significant differences between groups were tested using the Mann–Whitney method and are represented (*p* < 0.05).
(TIF)

S2 Fig Levels of IL-22, IFN-γ, IL-12p70, IL-13, IL-17A, TNF-α and IL-4 in plasma in children with symptomatic uncomplicated malaria, moderate malarial anemia and severe malarial anemia. Their plasma concentrations were quantified using enzyme-linked immunosorbent assays (ELISA) and compared between the different groups in pairs. Cytokine concentrations were represented by boxplots with medians and interquartile range (IQR) in pg/mL on the log10 scale. Statistically non-significant differences between groups were tested using the Mann–Whitney method and are represented (*p* > 0.05).
(TIF)
