# Supplementary figures and images for: Cytokine response in asymptomatic and symptomatic Plasmodium falciparum infections in children in a rural area of south-eastern Gabon
Source: PLoS One. 2023 Feb 14;18(2):e0280818. doi: 10.1371/journal.pone.0280818 (PMC9928122; doi:10.1371/journal.pone.0280818)

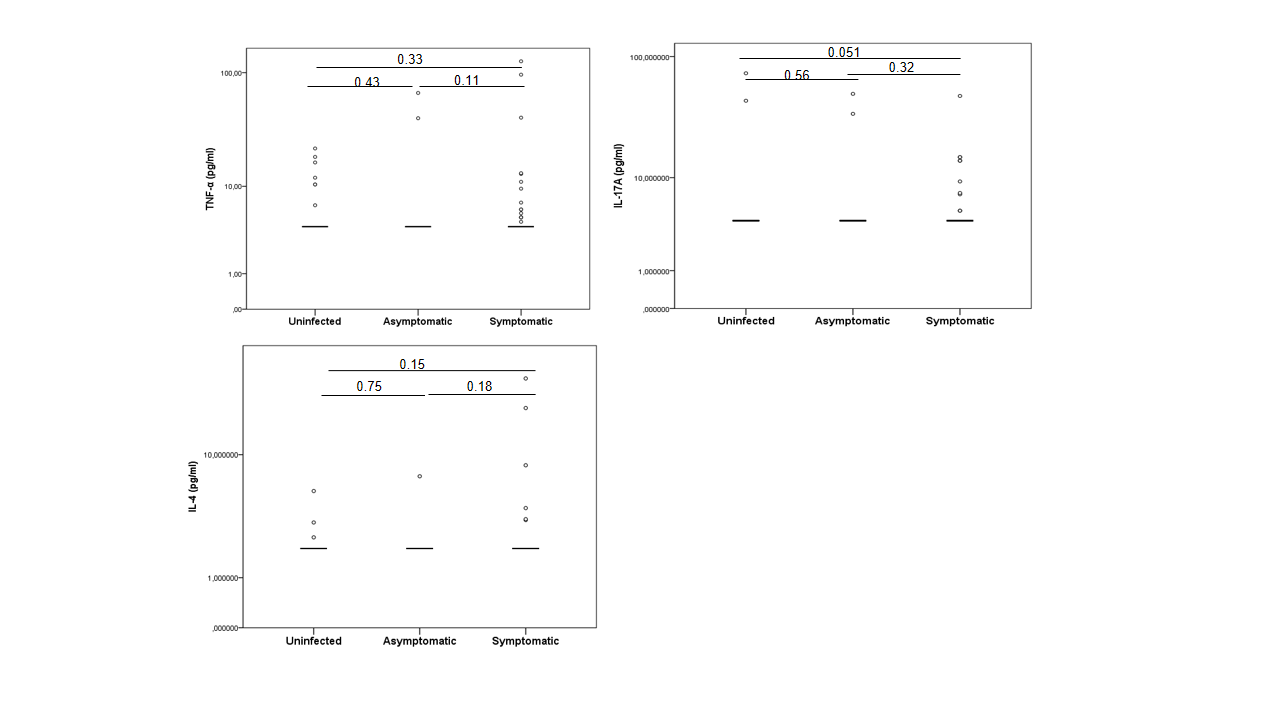

Supplement: S1 Fig — Circulating levels of each in the plasma were compared between uninfected, symptomatic and asymptomatic children in pairs. Concentrations were quantified using enzyme-linked immunosorbent assays (ELISA). Cytokine concentrations were represented by boxplots with median and interquartile range (IQR) in pg/mL on the log10 scale. Statistically significant differences between groups were tested using the Mann–Whitney method and are represented (p < 0.05). (TIF) [file pone.0280818.s001.tif]

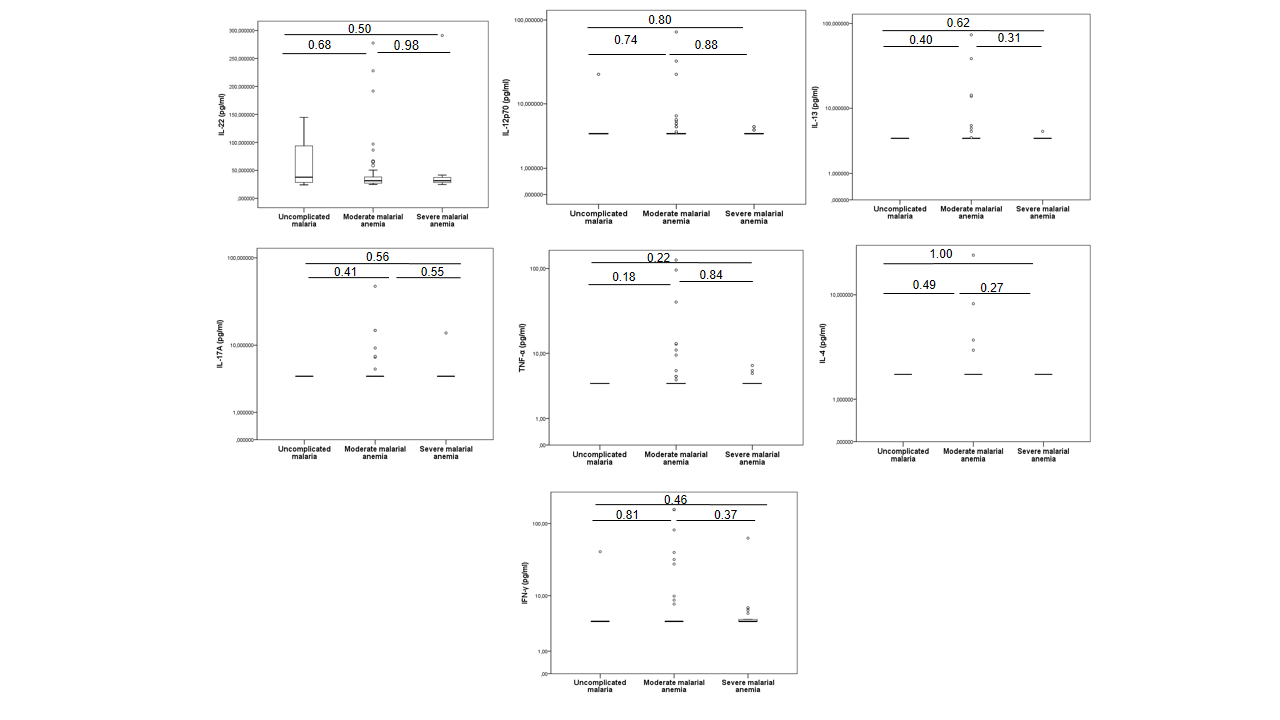

Supplement: S2 Fig — Their plasma concentrations were quantified using enzyme-linked immunosorbent assays (ELISA) and compared between the different groups in pairs. Cytokine concentrations were represented by boxplots with medians and interquartile range (IQR) in pg/mL on the log10 scale. Statistically non-significant differences between groups were tested using the Mann–Whitney method and are represented (p > 0.05). (TIF) [file pone.0280818.s002.tif]
